# Knowledge transfer: what drug information would specialist doctors need to support their clinical practice? Results of a survey and of three focus groups in Italy

**DOI:** 10.1186/s12911-016-0355-7

**Published:** 2016-09-01

**Authors:** Giulio Formoso, Paolo Rizzini, Maurizio Bassi, Paolo Bonfanti, Giuliano Rizzardini, Annalisa Campomori, Paola Mosconi

**Affiliations:** 1 Pharmaceuticals and Medical Devices Area, Health and Social Policies Directorate, Emilia- Romagna Region, Bologna Italy; 2 ViiV Healthcare, Verona, Italy; 3 Fondazione Smith Kline, Verona, Italy; 4 Department of Infectious Diseases, Provincial Hospital Authority, Lecco, Italy; 5 Department of Infectious Diseases, Luigi Sacco Hospital, Milan, Italy; 6 Hospital Pharmacy, Provincial Authority for Health Services, Trento, Italy; 7 Laboratory of Citizen Involvement in Health Care, IRCCS Mario Negri Institute of Pharmacological Research, Milan, Italy

**Keywords:** Drug information, Knowledge transfer, Evidence-based medicine, Information validity, Conflicts of interests, Information sources, Information tools

## Abstract

**Background:**

The wide offer of information on pharmaceuticals does not often fulfill physicians’ needs: problems of relevance, access, quality and applicability are widely recognized, and doctors often rely on their own experience and expert opinions rather than on available evidence. A quali-quantitative research was carried out in Italy to provide an overview on information seeking behavior and information needs of doctors, in particular of infectious disease specialists, and to suggest an action plan for improving relevance, quality and usability of scientific information.

**Methods:**

We did a quantitative survey and three focus groups. Two hundred infectious disease specialists answered a 24-item questionnaire aimed at investigating features of scientific information they receive and their ratings about its completeness, quality and usability. Subsequent focus groups, each involving eight specialists, investigated their opinions on information sources and materials, and their suggestions on how these could better support their information needs.

**Results:**

The quantitative survey indicated doctors’ appreciation of traditional channels (especially drug representatives) and information materials (brochures), but also their attitude to autonomous search of information and their wish to have more digital channels available. Focus groups provided more depth and, not surprisingly, revealed that physicians consider critical to get complete, comparative and specific information quickly, but also that they would like to discuss their doubts with expert colleagues. Quite strikingly, limited concerns were expressed on information validity, potential biases and conflicts of interests, as scientific validity seems to be related to the perceived authoritativeness of information sources rather than to the availability of a transparent evaluation framework.

Although this research investigated views of infectious disease specialists, we believe that their opinions and perceived needs should not substantially differ from those of other clinicians, either in primary or in secondary care.

**Conclusions:**

In participants’ view, the ideal information framework should provide quick and tailored answers through available evidence and favor the exchange of information between practitioners and trusted experts. The general consensus existing within the scientific and medical community on the need for integrating available evidence and experience is confirmed, although the issues of information validity and conflicts of interests seem definitely overlooked.

**Electronic supplementary material:**

The online version of this article (doi:10.1186/s12911-016-0355-7) contains supplementary material, which is available to authorized users.

## Background

### The information paradox

This definition has been used a couple of decades ago to qualify a situation health professionals often face: loads of information from clinical studies, reviews, guidelines made available through journals, websites, meetings, etc. but not matching with access to valid, relevant and eventually useful information when it is needed [1, 2]. In two decades this situation has not substantially changed: the information flow is even growing, considering the ease of diffusion of digital sources and their increasing popularity among physicians, [3] so that in a recent editorial from the New York Times physicians are attributed a “Stendhal syndrome”, such as the French writer who felt overwhelmed by the pieces of art surrounding him when he visited Florence [4]. However, unlike the relevance (and beauty) of Florentinian paintings and sculptures, information materials targeted at practitioners less frequently offer relevant and also intelligible, practical and unbiased information, free from conflicts of interests (CoI), on the effectiveness and safety of drugs and other health care interventions that can be usefully transferred to clinical practice [5, 6]. The evidence-based medicine (EBM) movement advocated the integration of individual clinical expertise with the best available external clinical evidence from systematic research [7]. However, difficulties in accessing and evaluating such evidence and limits in its application to individual patients [8] may cause over-reliance on expertise, either as own experience or as enlightenment from “experts”, [9] and on information brought directly or indirectly by drug companies; the latter often represents physicians’ main information source but inevitably reflects interests of drug producers, [10] often being associated with higher prescribing frequency and costs or lower prescribing quality [11].

The gap between doctors’ information needs and access to valid and relevant information does not seem a fully exploited field of research. Specifically, there is limited research on doctors’ opinions about pros and cons of different information sources, about how “old” channels (like pharmaceutical sales representatives – PSR - and seminars) and materials (like scientific papers and flyers) can be integrated by “new” digital channels (like websites, tablet apps, e-mail alerts, etc.) to provide physicians with relevant information and fill the knowledge gaps. In this regard opinions of hospital specialists may be of particular relevance, considering their strong request of information of high quality, often under time pressure. The area of infectious diseases is of specific interest, as in the last 15–20 years it witnessed a sharp improvement in effectiveness of therapies, in particular in the HIV area, but also potential concerns about their high costs and consequently their sustainability for health systems: for these reasons, physicians in this clinical area may provide relevant insights about the added value of drug information sources.

This quali-quantitative research explored opinions and suggestions of infectious disease specialists to provide an overview of information seeking behavior and information needs of doctors, to suggest an action plan to improve quality, relevance and usability of information on drugs, the latter representing a key “equipment” for both secondary and primary care physicians.

## Methods

The research has been supervised by an Advisory Committee and has been carried out by Doxa Institute, an opinion research company, Italian branch of Gallup International Association. It consisted of two parts: a quantitative survey and a qualitative analysis by means of three focus groups.

- Quantitative survey. In September 2014, two hundred infectious disease specialists expert in HIV therapies were involved through Computer Assisted Telephone Interview (CATI) and asked to answer to a 24-item pre-tested questionnaire (see Additional file 1). This survey was aimed at describing: the type and frequency of drug information they receive from drug firms or that they independently seek; investigating their preference (expressed through numeric scales from 1 to 10) about specific channels and features of scientific information they receive from PSR, meetings/congresses, courses, e-mails/web calls and from independent sources; asking their ratings about the quality, completeness, usability and credibility of such information, about which methodological characteristics of the presented data may affect the quality of information and about their preference on traditional vs new digital information sources; and finally describe their unmet needs. The questionnaire was pre-tested on ten physicians to assess content validity and comprehensibility. The final sample was randomly selected through an automatic system of management of phone interviews, to allow for a balanced distribution of respondents coming from four geographic areas of Italy (north-west, north-east, centre, south and islands). The sampling frame came from a list of about 1000 physicians, whose names were collected by Doxa, working in infectious diseases hospital wards within the Italian NHS or in NHS accredited hospitals, specialized in providing care to HIV patients. A 95 % confidence interval of ± 6,2 % was associated with the sample size chosen. Data check and descriptive statistics were performed using SAS® and DIANA® statistical software, respectively.
- Qualitative research. Three focus-groups were run in March 2015 in different cities (Milan, Rome and Naples) and geographical areas, each involving eight infectious disease specialists - expert in HIV therapies - to discuss more in depth their information needs in the context of clinical practice, their experience with different information channels, and their suggestions on how they could be better supported in order to meet their information needs. Doctors were asked to share their opinions on drug information starting from their view of the current model, exploring their information needs and stimulating their suggestions on optimal ways to address these needs. Specifically, the moderator progressively elicited their opinions on information sources and materials as for their reliability (scientific validity and transparency), completeness (if there are gaps in information they would need and if they are presented with all the available information) and usability (easy access, rapid uptake and implementability). Additional file 2 provides details about the structure of the focus groups and their contents, addressed through specific questions. Participants were randomly selected out of the same list mentioned above as for the survey methodology; criteria were adopted to avoid that more than two of them worked in the same hospital. None of the selected physicians had participated to the quantitative survey. Participants were not reimbursed for their time, but provided with petrol coupons so that there were not at a financial loss to attend the focus groups in person. Focus groups were led by an expert moderator. Participants’ answers were analyzed using content analysis, by creating sub-categories and verifying the association between the formulated hypotheses and contents emerging from the focus groups. Quotes of specific phrases stated by participants have been reported (in italics); each participant was given an unique ID code, assigned consecutively in each of the three focus groups (ID 1 to 8, 9 to 16 and 17 to 24), to allow assessing the spread of participant opinions.

## Results

- Quantitative survey
  To obtain a geographically balanced sample of 200 respondents, 364 physicians had been contacted; 146 (40,1 %) declined to participate and 18 (4,9 %) did not comply with the sampling criteria. Respondents were mostly males (71 %), well experienced professionals (only 17 % had less than 15 years of professional experience) mostly assisting more than 300 patients a year (Table 1). In the previous 4 months almost all of them (98 %) received information from PSR, on average a couple of times a week; more than one-third also got in contact with PSR through phone or web calls. Sixty-five percent said they had participated to sponsored meetings/congresses.

**Table 1:** Characteristics of survey respondents (*n* = 200)

Geographical area	Percent
•North west	27 %
•North east	15 %
•Center	25 %
•South	33 %
Sex	
• male	71 %
• female	29 %
Age	
• ≤35	14 %
• between 35 and 45	13 %
• between 46 and 54	37 %
• >55	49 %
Professional experience	
• ≤15 years	17 %
• between 16 and 20 years	21 %
• between 21 and 25 years	26 %
• >25 years	36 %
Assisted patients/year	
• ≤100	16 %
• between 101 and 300	32 %
• >300	52 %
  Generally speaking, PSR were the most appreciated *channel* of information (average score of 8.5 out of 10) followed by sponsored meetings/congresses (7.8/10). Web calls (7.4/10) and especially phone calls (6.5/10) were quite less appreciated, indicating doctors’ preference for information received through contacts face-to face (Table 2). More specifically, PSR were the most appreciated channels in terms of quality, credibility, completeness and usability (score 8.5 or 8.6/10) although responses show little discrimination among different channels, including those independent from drug industry (Table 3). Opinions about transparency and awareness of CoI followed the same pattern, and PSR were regarded as the most transparent channel (score: 8/10). As for qualifying contents presented by PSR, respondents also showed little discrimination among specific characteristics (like availability of RCTs or systematic reviews, or use of absolute risk differences, or detailed information about patient characteristics, etc.) and their perceived importance (Table 4).

**Table 2:** Respondents’ scores on importance of information channels (scale: 1–10; *n* = 200)

	Average score	% scoring at least 8/10
Pharmaceutical sales representatives	8.5	92 %
Sponsored meetings - congresses	7.8	73 %
Sponsored CME courses	7.5	68 %
E-mail from drug companies	7.4	64 %
Web-calls with pharmaceutical sales representative	7.4	58 %
E-mail from journal	7.3	59 %
Phone calls from pharmaceutical sales representative	6.5	30 %

**Table 3:** Respondents’ average scores on specific attributes of information channels (scale: 1–10; *n* = 200)

	Quality	Credibility	Completeness	Usability
Pharmaceutical sales representatives	8.6	8.5	8.5	8.5
Sponsored meetings - congresses	8.3	8.2	8.2	8.1
Sponsored CME courses	8.2	8.2	8.1	8.1
Information independent from drug industry	8.3	8.3	8.3	8.3

**Table 4:** Respondents’ scores on importance of specific elements in information brought by pharmaceutical sales representatives (scale: 1–10; *n* = 200)

	Average score	% scoring at least 8/10
Availability of RCTs vs placebo	8.1	81 %
Use of absolute risk differences	8.0	79 %
Availability of RCTs vs best alternative treatment	8.0	77 %
Detailed information about patient characteristics	8.0	76 %
Availability of systematic reviews	7.9	75 %
Availability of RCTs not sponsored by drug companies	7.9	74 %
Indication of the added value of drugs under study	7.9	71 %
  As for scientific and/or promotional *materials*, most respondents (89 %) received brochures from PSR, although two-thirds also got paper information available through websites and 29 % got kit of slides or multimedia files. Overall, materials received were mostly (75 %) in traditional (paper) format. Brochures were the preferred ones (by 38 % of respondents) followed by information available through websites (20 %). Although opinions about traditional materials and channels of information were quite positive, the possibility of “digital” innovation (for example, receiving more information through the internet or digital apps) was regarded favorably and 70 % would have preferred digital channels, if available.
  Overall, 84 % of respondents said they are satisfied with information received (no unmet needs) and just 8 % said they would like more information on a regular basis, but 71 % declared that they often or always search information autonomously, especially through the web and through scientific literature (for 42 and 20 % the preferred sources, respectively).
- Qualitative research
  We report qualitative findings highlighting participants’ quotes in italics, associated with participants’ ID codes (see also Table 5).

**Table 5:** Some quotes from focus groups on participants’ opinions about key features of drug information

Feature	Quotes
Reliability	*“… information is reliable when it is transmitted by an authoritative source …”*
	*“… world-famous opinion leaders … big shots, who have great experience and have done pivotal studies … when they talk, we just listen …”*
	(in scientific research) *“… the presence of authoritative and independent researchers is important …”*
	(in scientific journals) *“…drugs can be evaluated by researchers outside the sponsor company …”*
	*“… if the goal is improving patient care I trust it … I don’t if we talk about piddling …*
Completeness	*“… it’s necessary to look at everything … let’s not just look at positive data … every drug has weaknesses and knowing about them would bring added value to info …”*
	*“ … comparative information to understand which drug is better for one patient and which for another one …”*
	*“… if you don’t relate information to everyday practice, what is the use of it?…”*
Usability	*“… easily accessible, through the web … in Pubmed … possibly free of charge …”*
	*“… not too detailed … or pompous … it should be strict to the point … providing hints that I can deepen on my own …”*
	*“… when I ask for information, drug reps bring it to me the day after …”*
	*“… I like information targeting specific needs … personalized …”*
	(guidelines refer to populations) *“different from your own patients … in different countries they say different things … you cannot read them in one minute … they are huge …”*
Exchange	*“… I would like talking with colleagues … they may have more experience with specific types of patients … sometimes I don’t know how to manage them… I’d like getting help to choose the right drug for each patient …”*
	*“… people with field experience … with knowledge that you can’t find anywhere …”*
	*“… information-sharing is really enriching … it creates synergy … it is quick, maybe directly at bedside … it refers to real situations, unlike statistics in congresses …”*
	*“… let’s talk about real cases … there is a wide array of patients … they are different from theory … we also cure immigrants …”*
Transparency – conflicts of interest	(drug reps) *“… you know which side they are on and they are honest in admitting that …*”
	(sponsored symposia) *“… it is clear who is behind them … knowing it, we don’t have any problem …”*

### Pharmaceutical sales representatives and sponsored sources

Positive opinions about PSR were confirmed. Flexibility in setting meeting times and helpfulness in providing specific information “on demand” look as the main reasons for such appreciation: *“… when I ask for information, PSR bring it to me the day after” “I like information targeting specific needs … personalized”* [participant ID (P) 20]. In this specialist setting, respondents considered PSR well prepared and able to put the information critically in context (*“… they compare their drug to other ones, bringing bibliography to support it”* [P 1]). Transparency of PSR goals was also considered very favorably: the objectives of PSR and in general of the pharmaceutical industry are explicit and knowing agenda and CoI of information providers was regarded as a very important feature *(“… you know which side they are on and they are honest in admitting that*” [P10]). For this reason, also sponsored symposia were deemed transparent and therefore not problematic (*“It is clear who is behind them … knowing it, we don’t have any problem”* [P2]); usefulness of symposia lies in the possibility of integrating different points of view, also with the contribution of specialists in different areas *(“… for example, you can talk with urologists to see how they approach infectious diseases in urology”* [P11]), although time required to participate was considered an important limit (*“… you can only choose few events a year … depending on the time you can put on them”* [P12]). Activities sponsored by foundations associated to drug companies were instead perceived as *“… more problematic, as their goals are not transparent”* [P15]. They were perceived as having a potentially relevant role though, as *“…arbiters between pharmaceutical companies”* [P13], “… *setting the field for objective, comparative assessments”* [P14] “… *providing trustworthy information … being providers of culture”* [P22]; in this regard, they should not be related to (financed by) one specific company, so that their neutrality would be assured. CME courses, providing credits for professional update, often organized by pharmaceutical companies, were perceived as the least useful information channel (*“… a lost occasion to get relevant updates”* [P8] “… *completely useless … just window-dressing … nobody checks if their contents are adequate”* [P3] “… *we are just forced to participate to get professional credits”* [P10]).

### Other information channels

As for channels not directly associated with pharmaceutical companies, scientific societies were highly regarded as they *“… promote the scientific debate”* [P6] *“… using authoritative sources, like world-famous opinion leaders … big shots, who have great experience and have done pivotal studies”* [P8] “… *when they talk, we just listen”* [P24]. Reliability of scientific journals (in particular of those perceived as the most relevant ones) was highly trusted too, since information is peer-reviewed (*“…drugs can be evaluated by researchers outside the sponsor company”* [P19]) and their online availability was seen as a great advantage, allowing relatively easy searches of different articles on the subject of interest and providing a wide array of information on drug effectiveness and safety (*“… they are easy to search, using the right keywords”* [P14]). In this regard, internet in general was considered as a wide and potentially relevant reservoir of information (*“… you can find everything you need … just in time”* [P14] “… *have an overview”* [P12] “… *on new drugs, drug interaction, safety”* [P14]*)* but attention is needed in selecting trustworthy information (*“… there is a risk of finding false information”* [P21] *“… you must be careful”* [P12, P21]). Lastly, guidelines (mostly those developed by international panels) were considered potentially useful for presenting a wide array of information *(“… they may not tell you the right solution for a specific patient, but can help make your way”* [P12] *“… and tailor one”* [P21]), although they often refer to populations *“different from your own patients*”, [P13] may reflect the context of the country where they have been written (*“… in different countries they say different things”* [P19]) and are time consuming (*“.. you cannot read them in 1 min”* [P12] “… *they are huge”* [P2]). On the other hand, expert opinion and word of mouth among colleagues (*“… people with field experience”* [P14] *“… with knowledge that you can’t find anywhere”* [P22]) was pointed as an extremely important information channel (*“… information-sharing is really enriching”* [P10] *“… it creates synergy”* [P11] *“… it is quick”* [P4] *“… maybe directly at bedside”* [P23] “… *it refers to real situations, unlike statistics in congresses”* [P4]).

### Qualifying characteristics of scientific information

In participants’ view, reliability of information especially depends on the presumptive authoritativeness of the source (*“… information is reliable when it is transmitted by an authoritative source”*) [P16] and of researchers submitting it (*“… the presence of authoritative and independent researchers is important”* [P14]) rather than on critical assessment of evidence; other than that, reliability was associated with relevance to patient care (*“… if the goal is improving patient care I trust it … I don’t if we talk about futile arguments”* [P17]) and to items like study sample size (“… *sometimes they don’t even tell you about that*” [P5]) and comparison groups (*“… knowing the difference vs alternative treatments would be very useful”* [P7]).

Completeness of information was associated with the availability of negative and not just positive data (*“… it’s necessary to look at everything … let’s not just look at positive data”* [P3] “… *every drug has weaknesses and knowing about them would bring added value to info”* [P8]*),* of data on side effects and drug interactions, on comparative data to assess *“… which drug is better for one patient and which for another one”* [P8] and of post-marketing data so that effectiveness in everyday practice could be assessed *(“… if you don’t relate information to everyday practice, what is the use of it?”* [P5]).

As for usability, information should be *“easily accessible … in Pubmed”* [P18] *“… through the web, possibly free of charge”* [P9] and *“… not too detailed … or pompous … it should be strict to the point … providing hints that I can deepen on my own …”* [P6].

### How physicians’ information needs may be addressed

According to participants, a community of physicians coordinated by trusted opinion leaders and connected through the web could be the ideal answer to physicians’ information needs (*“… I would like talking with colleagues”* [P10] “… *they may have more experience with specific types of patients”* [P9] “… *sometimes I don’t know how to manage them”* [P19] “… *I’d like getting help to choose the right drug for each patient”* [P10]). Colleagues could share their case reports and their everyday experience (*“… let’s talk about real cases … there is a wide array of patients … they are different from theory … we also cure immigrants”* [P11]), which could be integrated by easily accessible evidence-based information from bibliographic databases and authoritative scientific journals.

## Discussion

This research offers a number of important elements to enlighten the debate on doctors’ information needs and information seeking behavior, integrating the results of a quantitative survey with findings from three focus groups, and gathering participants’ suggestions on how to improve access to, perceived quality, relevance and usability of, scientific information. In particular, focus groups enriched findings from the quantitative survey, allowing for better discrimination of perceived reliability and usefulness of different information sources, for in-depth analysis of physicians’ information needs and for defining their suggestions to meet these needs. Even considering the specificity of investigating opinions of specialists in infectious diseases and HIV therapies in Italy, which is the main limit of this research, we believe that it could bring relevant contents to the debate on drug information needs in general as drugs are the most important “equipment” for all clinicians - in either secondary or primary care - and all of them share the need to keep pace with drug related research in spite of hurdles like time constraints, pressure from drug companies and also from patients and health managers.

The quantitative survey showed that PSR are considered as valuable information providers, mainly because they are always available to supply information with a flexible schedule and also “on demand”. We cannot say whether such views were swayed by other positive reinforcements offered by PSR in addition to their support and time flexibility. However, appreciation of “traditional” channels (PSR) and info materials (brochures) seemed to be partly contradicted by doctors’ positive attitude to autonomous search of information, and by their wish to have more digital channels available to build an enhanced information framework; anyway, the overall picture emerging from the survey suggests that respondents were quite satisfied with the information they receive. Focus groups allowed for in-depth analysis and integration of these initial findings, showing a more articulated picture and a deeper understanding of limits that currently available information may have. In particular, participants’ opinions about strength and weaknesses of different information sources, channels and materials clearly revealed two general features that an information framework should have in order to meet with doctors’ perceived needs: on one side, physicians would like to be able to get – from dedicated professionals like PSR and also autonomously – complete, comparative (vs alternative treatments) and practical information quickly, targeting specific needs they would have in specific clinical circumstances, so that it can be easily used in everyday clinical practice; on the other side they would need to discuss their doubts with expert colleagues and get support from their experience. Therefore information focusing on specific needs should be “quick and easy” to access through PSR, internet and digital supports but should be also, and especially, shareable among practitioners and mediated by expert opinion: in other words, an evolution would be needed towards a more integrated use of the web for quick and targeted answers to clinical questions, combining the power of bibliographic and clinical databases on one side and clinical experience and expert opinions on the other side.

These findings are not completely new. Looking at the available literature, even though scientific articles from a few years ago may not fully reflect the current situation with the evolution of information frameworks, there are two main elements that characterize the information needs of doctors and their seeking behavior. First, their demand for quick answers associated to time constraints; [12–14] and second, their reliance on experience from expert colleagues, who can (quickly) provide hints overcoming the difficulty to apply information about “average” patients to their own clinical encounters [2, 12, 15].

It is worthy of note that participants to our research were primarily concerned with the object of the information they want (targeted to specific patient needs - often not covered in scientific literature, which is mainly centered on “average” patients) and on the information framework that should provide it quickly (hyper-connected, with both expert colleagues and databases). Limited concerns were expressed on more specific characteristics of information, like its validity, potential biases and actual “evidence-base”: the need for information materials based on a reproducible analysis of the available evidence (therefore, based on a systematic methodology) was not focused on, and the threats due to non-publication of negative studies (publication bias) were generically dealt with as need to have also negative and not just positive data available. Limited concerns were also expressed about the need for information independent from vested interests, although independence – together with authoritativeness – of researchers has been cited as desirable. In this regard, knowing who produces the information was generally regarded as a fundamental element to qualify it as potentially useful, regardless of the presence of CoI. For example, participants did not show concerns about possible CoI and hidden biases associated to sponsored studies: they were confident that the filter operated by peer-review in scientific journals helps select valid research and that their own evaluation, together with their own and their colleagues’ personal experience, could help identify scientifically valid information as well as its applicability. Scientific validity seems to be assured by the perceived authoritativeness of information sources rather than the availability of an explicit and transparent evaluation framework, which is not asked for. Journal names, or names of experts who share their opinion, or names of scientific societies seem more important than an evidence-based assessment of the contents, that information materials generally do not provide in a plain and intelligible way and which would require time and skills that doctors generally do not have [16, 17]. No concerns about CoI of scientific journals [18] had been expressed either.

If we consider qualitative limits in the current offer of information to physicians, lack of EBM training in medical schools and time constraints, relying on the perceived authoritativeness of experts, local opinion leaders, [19] journals or societies may be an expected outcome and may often be a necessary shortcut to overcome difficulties in assessing the available evidence and using it in everyday practice. Risk of slipping from evidence to “eminence” based medicine – as self-referential assessment of health care interventions has been facetiously defined [20] - is concrete. More efforts are needed to provide doctors with more informative, useful and, at the same time, scientifically valid materials (either on web or on paper), based on systematic analyses of the available evidence [21] and synthesized through easy-to-read formats. These should ideally show a clear description of the characteristics of study populations, discuss results using absolute risk differences, evaluate possible study biases and highlight what the information does add in defining the place in therapy of drugs comparing to what is known about the therapeutic alternatives [5, 22]. Of course, web materials would allow different and additional layers of information through the use of hypertexts, and enhanced search engines would help accelerate the search for tailored answers.

Such ideal framework for evidence-based information would not be at odds with experience and expert opinions mediating its translation in clinical practice: evidence itself, if deployed by a thorough evaluation of the clinical context where it would be applied, is perceived as an academic exercise and can be of little use. The need for integrating a transparent assessment of evidence with experience is what the evidence-based movement itself has always advocated as David Sackett, considered one of the fathers of EBM, clearly wrote in a landmark 1996 editorial: “*The practice of evidence based medicine means integrating individual clinical expertise with the best available external clinical evidence from systematic research”* [7]. In keeping with this principle, clinical practice guidelines are (or should be) developed through a systematic assessment of the available evidence [23] made by multidisciplinary groups of experts [24]. Such integration of evidence and experience, taking the collaborative nature of medical work into consideration, [25] is quite different from that “eminence-based” medicine, based on self-referential attitudes that many opinion leaders often show: while providing practical answers and hints to their colleagues, the so called experts would need to support their opinions with a transparent assessment of the available evidence, and show that they do not have potential CoI that may drive their opinions. This principle has been advocated for the development of clinical guidelines too, asking for clear disclosure of CoI and exclusion of experts with relevant and non-resolvable CoI [24].

## Conclusion

This research suggests that institutional subjects who want to provide information to physicians should answer to a big challenge: providing quick, tailored and patient-centered answers through a transparent framework of available, comparative evidence, assessed for its validity and referred to the clinical context where it would be used, mediated by trusted experts with no relevant CoI who could help put the evidence in context; and favoring the exchange of information between practitioners. A big, big challenge, whose complexity had been already highlighted 20 years ago in a landmark paper by Richard Smith, where he weighted the “evergreen” role of experience from colleagues: [2]*“… Probably there will be no single tool but a family of tools, and I suspect that, whatever sophisticated tools may be developed, the major source of information will remain colleagues*”. Transparency of information (in its selection and assessment) and of experts’ CoI will be essential elements to consider for anybody who wants to compete on this field. As for specific key features, the development of evidence-based but uncomplicated information tools is already in a quite advanced stage in some countries like for example UK, Germany, Canada and the US. However, the development of relevant policies on CoI seems to be a general problem [26, 27]. Not to forget that patient-centered answers will require more patient-centered research, [28, 29] as our focus groups clearly indicated: participation of patients’ representatives in multidisciplinary panels defining research priorities [30] is an upstream requisite, so that information is not built on insubstantial grounds.

## Additional files

Additional file 1: Questionnaire. Description of data: 24-item questionnaire used for the quantitative survey. (RTF 371 kb) Additional file 2: Topic guide for focus groups. Description of data: details about the structure of the focus groups and their contents, addressed through specific questions. (DOC 83 kb)

## Abbreviations

CoI: Conflicts of interests
P: Participant
PCS: Pharmaceutical sales representative
