# Supplementary material for: Knowledge transfer: what drug information would specialist doctors need to support their clinical practice? Results of a survey and of three focus groups in Italy
Source: BMC Med Inform Decis Mak. 2016 Sep 1;16(1):115. doi: 10.1186/s12911-016-0355-7 (PMC5007811; doi:10.1186/s12911-016-0355-7)
Supplement: Additional file 1: — Questionnaire. Description of data: 24-item questionnaire used for the quantitative survey. (RTF 371 kb) [file 12911_2016_355_MOESM1_ESM.rtf]

QUESTIONNAIRE


  	March 2014	


QUESTIONNAIRE NO.         |___|___|___|

Moderator: ______________________

(Resp. Code |__|__|__|__|__|__|)
	
Date of interview: |___|___| / |___|___| 2014      	


INTRODUCTION

Good morning/afternoon/evening, I am a moderator with DOXAPHARMA. We are carrying out a survey of INFECTIOUS DISEASES (ID) SPECIALISTS to find out about the type of information they receive about medicines. We would like you to help us by telling us your views and opinions. In accordance with section 13 of the Italian Privacy Law (Legislative Decree 196 of 2003), you can agree to take part in the interview.
The interview will take around 20 minutes, and I guarantee that any information that you give us will be treated in confidence and be completely anonymous.
A1
Yes, happy to take part immediately	1      GO AHEAD with E0
Yes, happy to take part, but not now	2      MAKE AN APPOINTMENT
NO, REFUSES	3     DOES NOT WANT TO BE CONTACTED 
	AGAIN 

1.	You are an … ? 

1.	HIV Specialist
2.	Other specialisation Thank and close

1a In your professional practice an ID Specialist, do you regularly treat HIV-positive patients?
1.	Yes
2.	NoThank and close

2.	On average, how many patients do you see per year? 

1.	Up to 100 patients
2.	Between 101 and 300 patients
3.	More than 300 patients

3.	Do you remember how many sales representatives you have received in the last fortnight?

|___|___| (Record the exact number)


4. On average, how many pharmaceutical products does each sales rep. present to you each time you receive him/her?

Products presented in-depth	|___|___| (Record the exact number)
Other products presented (product "recall")	|___|___| (Record the exact number)

5. Have any antiretroviral drugs been presented to you in the last fortnight?

1.	Yes
2.	No

(If Cod. 1 to question 5)
6. And do you remember how many antiretroviral drugs the sales rep. presented to you in the last fortnight?

|__|__|__| number of antiretroviral drugs presented in the last fortnight

7. What type of information have you received from the drug sales reps. in the last fortnight? How important is each type of material you received in your professional practice? Please use a 10-point scale where 1 = not at all important and 10 = extremely important.
And of the material listed, which is your preferred type?

		YES	NO	Does not know, does not remember	Level of importance for your profession
Scores 1-10	Preferred support materials	
a	Information leaflet (brochure, visuals, etc.)	1	2	3	|__|__|	|__|	
b	Reprint of an article published in a magazine printed in English	1	2	3	|__|__|	|__|	
c	Article published in a magazine printed in Italian	1	2	3	|__|__|	|__|	
d	Other information products, such as monographs on specific drugs, editorials	1	2	3	|__|__|	|__|	
e	Reports from conferences and/or symposia	1	2	3	|__|__|	|__|	
f	Kit of slides, audio-visual materials on products or
other non-paper media	1	2	3	|__|__|	|__|	
g	Information taken from internet websites or portals	1	2	3	|__|__|	|__|	

8. And again with regard to the last fortnight, have you received any information other than the types that I have just listed? If yes, which?

_____________________ __________________________________________________________


9. More specifically, at the last meeting, did you receive/were you given any explanatory materials from/by the sales rep.?

1.	Yes
2.	No

(If cod. 1 to question 9)
10. How was it presented to you?

1.	Printed materials
2.	On an iPad 
3.	Other (please specify) __________

11. Is a copy of the material (if printed) left with you or made accessible via the internet or on electronic media (if on an iPad)?

1.	I am left a copy of the printed materials
2.	The material is made available via the internet or on electronic media 

12. Now please think back over the last four months. Over this period via channels have you received materials about antiretroviral drugs that you consider promotional? How important for your profession are each of the following information channels? Please use a scale of 1 to 10, where 1 = not at all important and 10 = extremely important.  And again using a scale of 1 to 10, where 1 = not at all transparent and 10 = extremely transparent, how transparent about possible conflicts of interest did you find the promotional information about antiretroviral drugs you received via each of the following channels? 

		YES	NO	Does not know, does not remember	Level of importance for your profession
Scores 1-10	Level of transparency of information about a possible conflict of interest
Scores 1-10	
a	Sales rep. call 	1	2	3	|__|__|	|__|	
b	Sales rep. web call	1	2	3	|__|__|	|__|	
c	Promotional symposium at a conference	1	2	3	|__|__|	|__|	
d	Single-sponsor CME course	1	2	3	|__|__|	|__|	
e	By phone	1	2	3	|__|__|	|__|	
f	Information by email sent directly by the pharmaceutical company	1	2	3	|__|__|	|__|	
g	Information by email from a publication	1	2	3	|__|__|	|__|	
h	Information via text message	1	2	3	|__|__|	|__|	
i	Ward meeting with pharmaceutical company representative	1	2	3	|__|__|	|__|	


13. Now we would like to understand how important for you the inclusion of the following factors in considering a call from the drug sales rep. to be of a HIGH QUALITY. Please use a scale of 1 to 10, where 1 = not at all important and 10 = extremely important. 

		Importance scores 110	
1	Availability of an RCT (randomised clinical study) against placebo	1   2   3   4   5   6   7   8   9   10 	
2	Availability of an RCT (randomised clinical study) comparing the drug with the best available alternative	1   2   3   4   5   6   7   8   9   10 	
3	Availability of independent RCTs (randomised clinical studies)	1   2   3   4   5   6   7   8   9   10 	
4	Availability of systematic reviews	1   2   3   4   5   6   7   8   9   10 	
5	In-depth data on the benefits and absolute risks of the drug (who many patients per  	1   2   3   4   5   6   7   8   9   10 	
6	In-depth information on the specific characteristics of the patients studied (or the probability that they have results that can be applied to may patients)	1   2   3   4   5   6   7   8   9   10 	
7	In-depth explanation of the added value compared with the available alternatives	1   2   3   4   5   6   7   8   9   10 	

14. In general, how do you judge the information on the drugs for HIV received from the sales reps with regard to the following factors? Please give a score for each of the following factors using a scale of 1 to 10, where 1 = very poor and 10 = excellent (If Does not know, put 999). 

		Opinion scores 110	
1	Quality	Very poor 1   2   3   4   5   6   7   8   9   10   Excellent	
2	Comprehensiveness (good quantity of information about efficacy and tolerability)	Very poor 1   2   3   4   5   6   7   8   9   10   Excellent	
3	Credibility	Very poor 1   2   3   4   5   6   7   8   9   10   Excellent	
4	Usefulness/Usability	Very poor 1   2   3   4   5   6   7   8   9   10   Excellent	

15. In general, in the HIV segment, what is your opinion of information about drugs you have received at product conferences sponsored by a sole pharmaceutical company in the last four months with regard to the following factors? Please give a score for each of the following factors using a scale of 1 to 10, where 1 = very poor and 10 = excellent (If Does not know, put 999). 

		Opinion scores 110	
1	Quality	Very poor 1   2   3   4   5   6   7   8   9   10   Excellent	
2	Comprehensiveness (good quantity of information about efficacy and tolerability)	Very poor 1   2   3   4   5   6   7   8   9   10   Excellent	
3	Credibility	Very poor 1   2   3   4   5   6   7   8   9   10   Excellent	
4	Usefulness/Usability	Very poor 1   2   3   4   5   6   7   8   9   10   Excellent	


16. In general, in the HIV segment, what is your opinion of information about drugs you have received at CME courses sponsored by a single pharmaceutical company in the last four months with regard to the following factors? Please give a score for each of the following factors using a scale of 1 to 10, where 1 = very poor and 10 = excellent (If Does not know, put 999).
 
		Opinion scores 110	
1	Quality	Very poor 1   2   3   4   5   6   7   8   9   10   Excellent	
2	Comprehensiveness (good quantity of information about efficacy and tolerability)	Very poor 1   2   3   4   5   6   7   8   9   10   Excellent	
3	Credibility	Very poor 1   2   3   4   5   6   7   8   9   10   Excellent	
4	Usefulness/Usability	Very poor 1   2   3   4   5   6   7   8   9   10   Excellent	

17. In general, in the HIV segment, what is your opinion of information about drugs you have received from independent information sources (regional drug bulletins, AIFA updates or national guidelines) in the last four months with regard to the following factors? Please give a score for each of the following factors using a scale of 1 to 10, where 1 = very poor and 10 = excellent (If Does not know, put 999). 

		Opinion scores 110	
1	Quality	Very poor 1   2   3   4   5   6   7   8   9   10   Excellent	
2	Comprehensiveness (good quantity of information about efficacy and tolerability)	Very poor 1   2   3   4   5   6   7   8   9   10   Excellent	
3	Credibility	Very poor 1   2   3   4   5   6   7   8   9   10   Excellent	
4	Usefulness/Usability	Very poor 1   2   3   4   5   6   7   8   9   10   Excellent	

18. And in general. thinking of the calls by sales reps as a whole, how satisfied are you? Please use a scale of 1 to 10, where 1 = not at all satisfied and 10 = very satisfied.

Not at all satisfied1   2   3   4   5   6   7   8   9   10   Extremely satisfied

19. Have you ever received information from a pharmaceutical company sales rep. other than face-to-face?

1.	Yes
2.	No

(If cod. 1 to question 19)
20. Via which channel did you receive this information?

1.	Web call
2.	Email or phone
3.	Other (please specify) ___________


(To all)
21. Doctor, thinking about traditional information received during the sales rep's call on the one hand and via the new digital information channels on the other, and imagining a line in which at one end 1 = traditional drug information and 10 = drug information via digital channels, where would you place your own preference? 

Traditional drug information 1 2 3 4 5 6 7 8 9 10 Drug information via digital channels

22. And thinking in general about the type of information your normally receive, regardless of the channel used, are there any unmet needs that could improve the quality and efficacy of the information? Can you give me any actual examples?

_________________ _______________________________________________________________

23. Finally Doctor, we would like to understand your habits when it comes to independently seeking information of a professional nature. 
Do you normally look for information yourself in order to meet a professional n need for information without waiting for the sales rep. to call, or, in general, for a pharmaceutical company to contact you?

1.	Always
2.	Often
3.	Rarely
4.	Never

24. And when you look for the information you need to meet your needs by yourself, which main channel do you use?  Which other channels do you use in addition to this? (record first mentioned and then others mentioned)

1.	International literature
2.	Cochrane Reviews
3.	Medical/scientific company documents/websites 
4.	Pharmaceutical company websites
5.	Regulatory agency websites
6.	Websites of public bodies that issue guidelines or HTA reports
7.	Search engines
8.	Independent bulletins
9.	Other (please specify _________)


RESPONDENT DESCRIPTION

A.	Gender 

1.	Male
2.	Female

B. 	Age

1.	Up to 34
2.	35-45
3.	46-54
4.	55 or older

C. Professional seniority

|__|__| years

D. Region in which based

1	  Piedmont	11	Lazio
2	  Valle d'Aosta	12	Umbria
3	  Lombardy 13	Abruzzo
4	  Liguria	14	Molise
5	  Friuli Venezia-Giulia	15	Campania
6	  Trentino Alto-Adige	16	Puglia
7	  Veneto	17	Basilicata
8	  Emilia Romagna	18	Calabria
9 	  Tuscany	19	Sicily
10	  Marche	20	Sardinia	

E	Place of residence

1.	Major city
2.	Smaller town

F	Number of inhabitants in city/town

1.	Less than 30,000 	
2.	30,000 to 100,000	
3.	100,000 to 500,000	
4.	More than 500,000.
