# Supplementary material for: Knowledge transfer: what drug information would specialist doctors need to support their clinical practice? Results of a survey and of three focus groups in Italy
Source: BMC Med Inform Decis Mak. 2016 Sep 1;16(1):115. doi: 10.1186/s12911-016-0355-7 (PMC5007811; doi:10.1186/s12911-016-0355-7)
Supplement: Additional file 2: — Topic guide for focus groups. Description of data: details about the structure of the focus groups and their contents, addressed through specific questions. (DOC 83 kb) [file 12911_2016_355_MOESM2_ESM.doc]

1. **Warm up**

Presentation of meeting objectives.

Discussion rules: expressing ideas freely, no censure/criticism.

Participants’ self-introduction:

- name
- age
- interests and other personal details to favor informality

*In conducing the meeting, the moderator must explore different areas related to information seeking behavior, perceived relevance of information and information needs about drugs, and can use the following series of questions/themes to elicit participants’ opinions*

1. **The current model of scientific information in the HIV area: scenarios and perspectives**

- If you think about scientific information in the HIV area, which are your first thoughts?
- What is the role/importance of scientific information in your professional activity?
- What can you say about scientific information as it is today? How is it changed/evolved?
- As for drug information, which SOURCES do you consider the most reliable?
- Why? Which elements make them the most reliable?
- Which sources do you consider the least important instead? Why?

*If they don’t come up spontaneously, the moderator elicits comments about*

*- medical societies*

*- independent foundations*

*- scientific societies*

*- PDR*

*- symposia*

*- CME courses*

- *In your opinion, which role and differences are there among these sources?*

*The moderator also tries to elicit participants’ reasoned opinions about validity, transparency, completeness, access and ease of use of different drug information sources, asking for practical examples.*

- Scientific information: which ELEMENTS make it reliable? Why?
- As for scientific information, what makes it “complete”?
- And what makes it “usable”?
- What do you think about PDR and about their role?
- Which elements should PDR take into account to provide reliable and effective drug information?
- Which is the value of information from PDR? Which information they provide is relevant?
- Which CONTENTS are provided through the various SOURCES of scientific information?

*The moderator encourages participants to think about contents provided by:*

*- medical societies*

*- independent foundations*

*- scientific societies*

- *PDR*

- *symposia*

- *Which differences among contents provided by these sources?*
- *Which differences in terms of value? In terms of relevance for clinical practice?*
- As for specific MATERIALS used to transfer drug information, which are the best ones? Why? Which are their advantages and disadvantages for clinicians?

*If they don’t come up spontaneously, the moderator elicits comments about:*

- - *Brochures*
  - *Papers in scientific journals (in original language)*
  - *Papers in scientific journals (translation)*
  - *Web pages*
  - *Interactive materials via tablet*
- Do you search for information autonomously? Why? What about it?
- As for your experience, how timely is the diffusion of drug information?
- Do you think there have been changes in scientific communication across the years? Which changes have been the most relevant?
- As for information materials, do you receive it more often on paper or through other tools?
- What do you think about new digital information channels/tools?
- What should be kept and what should be modified?
- Do you think that there will be changes in the way drug information is diffused? Why? How?
- Which are the positive aspects of drug information as it is today?
- What about the negative aspects?
- **Clinical studies:**
  - Which is the value/weight of clinical studies in helping your clinical practice? How?
  - Validity and reliability of clinical studies: how do you assess them? (sample size, selection criteria, etc)
  - Conflicts of interests: is there a problem considering the roles of sponsors, researchers, media where study results are reported?
  - In this regard, could an independent foundation have a relevant role in managing information on clinical studies?

1. **Information needs**

*The moderator asks each participant to write on single piece of papers (post-it) which needs they currently have on scientific information about drugs. These inputs from participants help to draw a list/map of information needs to favor debate among participants.*

- Which are the unfulfilled needs about drug information today?
- How do these needs arise?
- How have they been changing across time?
- In your opinion, what are the causes of this information gap?
- And what are their consequences for your professional activity?
- Can you make examples about suboptimal information on drugs?
- Which elements make drug information difficult/suboptimal?
- How do you address this problem?
- Do you share/discuss these difficulties with anyone? Who?
- How do you talk/what do you say about it?
- If you had to imagine an ideal framework of drug information, how would it be/which characteristics would it have?

1. **Creative phase: possible answers to information needs**

*The moderator splits the group in two subgroups, asking to think about possible answers to unfulfilled needs about scientific information on drugs. The goal is to develop an ideal model of scientific information. The moderator will then provide each subgroup with a poster to fill with participants’ proposals, collected within 3 boxes related to these themes:*

- WHAT: which kind of information?
- HOW: which channel?
- FROM WHOM: which sources?

*After collecting participants’ suggestions, the moderator will discuss with each group:*

- How your model can favor the provision of reliable, complete and usable information? Which elements can support it?
- Why did you suggest these sources?
- Why the channels you suggest would be better than other ones? How would it better fulfill your information needs?

1. **Solution building**

*A plenary discussion on the contents of the two posters concludes the focus group:*

- Considering the information needs you highlighted and the suggested solutions:
  - Analysis of ideas/suggestions to find a consensus on proposed models
  - Draw possible guidelines about a new model of scientific information on drugs:

- which specific actions could be implemented

- which ways/strategies could be added and improved to transfer information, and how

- How the proposed models could change the way scientific information on drugs is provided?
- Which of the models could be proposed/implemented by pharmaceutical firms and which ones by the National Health System (at local-national level)? Why?
- How could change your way to collect information?
- The moderator finally shows a few examples of printed information, developed through a social marketing approach[[1]](#endnote-2) and used in outreach visit programs (pharmacist meeting small groups of GPs),[[2]](#endnote-3) eliciting participants’ comments:
  - What do you think about this kind of information? About the way information is presented? How could it support your information needs and your clinical practice?
  - Which sources of information you consider reliable?
  - Considering this kind of information, who should deliver it? Why?

**Final thanks to the participants. Closure**

1. Formoso G, Marata AM, Magrini N. Social marketing: should it be used to promote evidence-based information? Soc Sci Med 2006;64: 949–53 [↑](#endnote-ref-2)
2. Magrini N, Formoso G, Capelli O, Maestri E, Nonino F, et al. Long term effectiveness on prescribing of two multifaceted educational interventions: results of two large scale randomized cluster trials. PLoS ONE 9(10): e109915. doi:10.1371/journal.pone.0109915 [↑](#endnote-ref-3)
